# The use of a surface active agent in the protection of a fusion protein during bioprocessing

**DOI:** 10.1002/bit.26817

**Published:** 2018-09-08

**Authors:** Peter Blas, Berend Tolner, John Ward, Kerry Chester, Mike Hoare

**Affiliations:** ^1^ Department of Biochemical Engineering ACBE University College London London UK; ^2^ Department of Oncology The Royal Free Hospital London UK

**Keywords:** degradation, fusion protein, pluronic acid, protection, surface active agent, USD shear device

## Abstract

The bioprocessing of a fusion protein is characterised by low yields and at a series of recovery and purification stages that leads to an overall 90% loss. Much of this apparent loss is due to the denaturation of a protein, missing a vital affinity ligand. However, there is evidence of the protection of degradation products which occurs in the presence of shear plus air/liquid interfaces. This study seeks out to characterise the loss and use ultra‐scale‐down studies to predict its occurrence and hence shows these may be diminished by the use of protective reagents such as Pluronic F68.

## 1 INTRODUCTION

It has been reported that a variety of proteins and enzymes are susceptible to conformational changes within a shear field (Biddlecombe et al., 2007; Charm & Lai, 1971; Charm & Wong, 1970; Charm & Wong, 1978). The production and purification of irregular and complex biopharmaceuticals exacerbates the problems a process engineer encounters during scale up (Junker, 2004; Kolade, Jin, Tengroth, Green, & Bracewell, 2015).

Many publications document that most biological entities are denatured or deactivated by high shear forces (Charm & Wong, 1970; Harrison, Gill, & Hoare, 1998; Lencki, Tecante, & Choplin, 1993; Levy et al., 1999). The recent advances in antibody‐based therapeutics, with fusion proteins for example (Michael et al., 1996), forces focus on the characterisation of complex protein structures during there interactions with harsh bioprocessing environments (Bowski & Ryu, 1974; Charm & Lai, 1971; Narendranathan & Dunnill, 1982; Virkar, Narendranathan, Hoare, & Dunnill, 1981; Tait, Hogwood, Smales, & Bracewell, 2012).

One way to achieve a better understanding could be the use of an ultra‐scale‐down (USD) shear device mimic. This device allows very small quantities of liquid to be inflicted to harsh hydrodynamic conditions, similar to what might be encountered during bioprocessing (Biddlecombe et al., 2007; Boychyn et al., 2001). Ultimately uncovering fundamental degradation properties of the biopharmaceutical being characterised. This could allow bioprocessing options to be studied at early stages of the development pathway in parallel to preclinical and clinical trials, thereby reducing the need for expensive pilot scale manufacture. The early prediction of robust and reliable large‐scale bioprocessing will allow speed to market, resulting in exclusivity and high profitability (Kolade et al., 2015; Rayat, Chatel, Hoare, & Lye, 2016).

Charm and Wong (1970) first documented that enzymes in a shear field lose activity. They showed that catalase, rennet and carboxypeptidase were all deactivated when subjected to shear stress (where no air bubble entrapment was occurring in the system), which was subsequently confirmed by Tirrell and Middleman (1975). However, other groups have suggested that shear stress does not have a significant effect if air–liquid interface does not exist (Thomas & Dunnill, 1979).

Work by various authors (Maa & Hsu, 1996; Thomas & Geer, 2011; Virkar et al., 1981) proposed that a shear stress environment without exposure to air–liquid interfaces has little influence on protein deactivation. When alcohol dehydrogenase was subjected to a high shear stress environment, little loss of activity was observed; however it was shown that secondary shear effects, for example, the addition of air in the system may lead to protein deactivation and/or aggregation (Virkar et al., 1981). Maa and Hsu (1996) created high shear rates of >10^5^/s, nevertheless it was found that a recombinant human growth hormone suffered little backbone clipping within a high shear environment, and no significant changes were found with recombinant human deoxyribonuclease.

These opposing arguments could be attributed to the presence of air–liquid interfaces in the shearing systems. It has been shown that the presences of air–liquid mixtures in large‐scale bioprocessing generate higher shear stresses, for example, the nonflooded and flooded feed zones in a pilot scale industrial centrifuge (Boychyn et al., 2001). Also, the variation in the opposing findings could be due to the different susceptibility of proteins with diverse structures in a high shear environment, further justifying the reasons for the present line of research. The aliphatic nature of proteins allows them to adhere to air interfaces (Damodaran., 2003), hence secondary shear effects like air–liquid interfaces maybe important in the way proteins aggregate or degraded (Maa & Hsu, 1997). Detergents like Pluronic^®^ (F68; Sigma‐Aldrich, Gillingham, Dorset, UK) are used in shear sensitive mammalian cell cultures with air–liquid interfaces to reduce aggressive high shear bubble detonation that would otherwise cause mammalian cell breakage (Chattopadhyay, Rathman, & Chalmers, 1994; Michaels, Petersen, Mclntire, & Papoutsakis, 1990; Tait et al., 2012; Tharmalingam, Wu, Callahan, & T. Goudar, 2015). Surfactants have also been used before to show that they have a protective effect on aggregation (Maa & Hsu, 1997). The additives used to reduce high shear at air–liquid interface in previous studies could be used in USD experiments to identify possible stabilising agents.

Irrespective of the mechanism of degradation of different proteins under shear stress can follow diverse types of degradation kinetics, theses include, pseudo first order (Pedley, Sharma, Hawkins, & Chester, 2003), and conventional first order (Harrison et al., 1998), second order (Lencki et al., 1993) and biphasic models (Lencki, Arul, & Neufeld, 1992a,b). Therefore detailed kinetic work in this field may elucidate important relationships of how specific proteins interact in a shear environment and could be used to predict protein degradation during larger scale bioprocessing (Rayat et al., 2016; Thomas & Geer, 2011).

This investigation focuses on the stability of an antibody‐fusion protein exposed to controlled levels of high shear typically found during large‐scale bioprocess operation, using USD shear device. The biopharmaceutical characterised in this study is a complicated fusion protein (MFECP1) used to treat colorectal cancer with a novel drug delivery system called, Antibody Directed Enzyme Prodrug Therapy (Bagshawe, 1989). The therapy works by targeting an enzyme carboxypeptidase CPG_2_ (42 kDa) to a tumour by virtue of its conjugation to a tumour specific‐antibody, MFE (27 kDa). After sufficient time for circulatory clearance, a nontoxic prodrug is administered. This prodrug is converted to a highly cytotoxic drug by the action of the enzyme at the tumour site (Begent et al., 1996; Michael et al., 1996).

By using millilitre quantities of this protein solution in a USD rotating disk shear device, it was possible to mimic the harsh conditions inflicted on the protein (Biddlecombe et al., 2007; Kolade et al., 2015; Levy et al., 1999).

The present research study has identified parameters that cause protein degradation and therefore the possible critical features that may elucidate the mechanism of damage that reduces yields. We report that air–liquid interface exacerbates the rate of fusion protein degradation. This information was further used to characterise how a shear protectant reduced breakdown profiles of the protein at large‐scale production improving the purified protein profile.

## 2 MATERIALS AND METHODS

### 2.1 Laboratory consumables

All laboratory consumables, plastic and glass were purchased from Fisher Scientific Ltd., (Leicestershire, UK) unless otherwise stated and were of the highest analytical grade.

### 2.2 Chemicals

All chemicals, unless otherwise stated, were obtained from Sigma Aldrich (Dorset, UK) and were of analytical grade. The following reagents were supplied by the Royal Free Hospital, Department of Oncology (London, UK), carcinoma‐embryonic antigen (CEA), NA1 (which is a functional domain of CEA), polyclonal anti‐CPG_2_ primary antibody raised in rabbit and anti‐MFE primary antibody.

### 2.3 Water for irrigation (WFI)

Sterile WFI (Baxter, Sigma‐Aldrich, Gillingham, Dorset, UK) was used in the fermentation production of the recombinant antibody‐fusion protein (MFECP1). Ultrapure deionised water (18.2 Ω; Milli‐Q System, Merck‐Millipore‐Sigma, Massachusetts, MA) was used for downstream purification steps and all USD shear experiments.

### 2.4 Fermentation of X33 *Pichia pastoris* with an expression of MFECP1

Production and purification of the fusion protein were carried out in the Academic Department of Oncology, Royal Free Hospital (University College London, London, UK). Fusion protein used in shear experiments came from Good Manufacturing Practice (GMP) Batch 81.

A fully accredited GMP fermentation protocol (Royal Free Hospital, London, UK) was used for producing X33 *P. pastoris* cells expressing the MFECP1 fusion protein with a C‐terminal hexahistidine tag (His6). The gene encoding the fusion protein was placed under an AOX1 promoter to allow methanol‐induced expression at 10‐L scale, described previously in detail (Tolner, Smith, Begent, & Chester, 2006a). The His‐tagged protein was captured and purified by expanded‐bed adsorption immobilised‐metal affinity chromatography (EBA) described in detailed (Tolner, Smith, Begent, & Chester, 2006b). After EBA capture the fusion protein fraction was concentrated and dialysed with Labscale^TM^ tangential flow filtration unit (Millipore, Merck‐Millipore‐Sigma, Massachusetts, MA) which was attached to a Pellicon XL 50 Biomax 30 (Merck‐Millipore‐Sigma, Massachusetts, MA) (30 kDa cutoff) ultrafiltration device. The final polishing step was purification by fast protein liquid chromatography. Twenty millilitre of concentrated and dialysed fusion protein was applied to a Superdex 200 column (GE Healthcare, Hatfield, UK) (GE Healthcare, Hatfield, UK) equilibrated with filtered phosphate‐buffered saline (PBS) mobile Phase 0.5 hr before loading at 0.4 ml/min. A consistent volume of protein between 250 and 300 ml was collected in 5 ml fractions and pooled giving 30 ml of purified protein yield from each fermentation. This fusion protein was used for shear experiments.

### 2.5 Storage of fusion protein

The purified fusion protein was frozen down into 1 ml aliquots and stored at −80°C for shear experiments at a later date.

### 2.6 Cell growth

To evaluate if F68 caused an effect on cell growth and/or expression of fusion protein, shake flasks were grown with different amounts of F68. A primary culture was prepared in a baffled shake flask, 0.25 L, containing 0.03 L of buffered glycerol‐complex medium which contained the following: yeast extract, 10 g/L; peptone, 20 g/L; potassium phosphate, 13.6 g/L; yeast nitrogen base (13.4%) 100 ml/L; biotin, 2 ml/L; glycerol 100 ml/L. This flask was then inoculated with 1 ml of MFECP1 seed lot from the GMP working cell bank (Royal Free Hospital) which was incubated overnight at 30°C and 250 rpm to an optical density (OD) of 6.0. On reaching an OD of 6.0, 5 ml of this primary culture was used to inoculate five shake flasks containing 0.025 L of methanol rich media buffered methanol‐complex medium which contained the following: yeast extract 10 g/L, peptone, 20 g/L; potassium phosphate, 13.6 g/L; yeast nitrogen base (13.4%) 100 ml/L; biotin 2 ml/L; methanol 5 ml/L and 0%, 0.01%, 0.1%, 0.5% and 1% F68 (vol/vol) respectively. The five flasks were incubated over a 96 hr period at 30°C and 250 rpm. The OD was monitored at an absorbance of 600 nm using the spectrophotometer. Hundred microlitre samples were taken at time points over the 96‐hr period, and their supernatants were frozen down for analysis at a later date.

### 2.7 Small‐scale liquid chromatography analysis

Samples from the higher concentration shear experiment (100 µg/ml) were analysed by small‐scale liquid chromatography to detect degradation fragments. 0.5 ml of each sample was loaded on to a 15‐ml small‐scale liquid chromatography column (Superose^TM^ 6 10/300 GL; GE Healthcare, Hatfield, UK) dimensions of the column were 10 x 300 mm. The mobile phase was filtered PBS and was pumped at a flow rate of 0.4 ml/min. The total run time was for 1 hr. The protein concentration was measured by optical absorption at 280 nm.

### 2.8 Assay methods

#### 2.8.1 CPG_**2**_ enzyme activity

Enzyme activity as described previously in Pedley et al. (2003), briefly the activity was defined where 1 U was equal to the amount of CPG_2_ enzyme required to hydrolyse 1 mmol of methotrexate per minute at 37°C. All enzyme activities were conducted in triplicates, Figure 8 show error bars as ± standard deviations.

#### 2.8.2 Sandwich enzyme‐linked immunosorbent assay (ELISA)

The ELISA used to analyse the MFECP1 fusion protein under investigation is described in detail in Pedley et al. (2003). Plates (NUNC Immunoplates Maxisorp; SLS, Thermo Fisher Scientific, Hvidovre, Denmark) were coated with NA1 (1 µg/ml) and incubated for 1 hr at room temperature. NA1 is the known functional domain on the CEA that interacts with a MFE‐23 antibody. Control wells were coated with PBS only under the same conditions. All wells were blocked with 5% milk proteins or PBS (150 µl/well) for 12 hr. Sheared fusion protein samples (100 µl samples) were applied and incubated for 1 hr. Detection of the intact fusion protein was carried out by incubating for 1 hr with polyclonal anti‐CPG_2_ primary antibody raised in rabbit, diluted 1/25,000 in 1% milk proteins/PBS (100 µl/well), followed by incubation with anti‐horse radish peroxidase (anti‐HRP) diluted 1/1,000 in 1% milk proteins/PBS (100 µl/well). Washing steps consist of four washes with 0.1% Tween 20/PBS (vol/vol), followed by three PBS washes. Plates were developed with *o*‐phenylenediamine in phosphate‐citrate buffer with sodium perborate (100 µl/well), and the reaction was stopped after 3 min with 4 M HCl, (100 µl/well). OD was measured at 490 nm on an Opsys MR ELISA plate reader (Dynex Technologies Ltd, Sussex, UK).

To calculate the approximate concentration of intact MFECP1 fusion protein in the sheared samples, a calibration curve was set up. Absorbances were measured at 490 nm of serial stock solutions from 500 to 31 ng/ml producing a calibration line giving a predictable relative error of ±10%. Calibration range from 700 to 500 ng/ml gave a ±20% relative error in calculating concentrations. Control experiments showed that the reagents F68 (0.01%, vol/vol) and antifoam (0.01%, vol/vol) gave a zero response at 490 nm.

#### 2.8.3 Sodium dodecyl sulfate‐polyacrylamide gel electrophoresis (SDS‐PAGE) and western blot analysis

Proteins were separated under reducing conditions by SDS‐PAGE on 12% Tris‐glycine gels (Thermo Fisher Scientific, Hvidovre, Denmark) at 125 V for 1.5 hr. Gels were then stained with Coomassie blue overnight, then de‐stained (21% MeOH; 8%, acetic acid; 71% H_2_O) and dried with gel drying solution (EtOH; Thermo Fisher Scientific, Hvidovre, Denmark) before mounting between plastic membrane. For the western blots proteins separated on the SDS‐PAGE gels were then transferred to polyvinylidene fluoride membrane (Bio‐Rad Laboratories Ltd, Watford, UK) at 125 mA for 90 min. For detection with specific antibodies, the membrane was blocked with 5% milk proteins (Marvel Milk powder, Premier Foods, St. Albans, Hertfordshire, UK)/PBS for 2–16 hr at 4°C. Immunoreactive detection of CPG_2_, fragment of the MFECP1 protein was performed by incubation with polyclonal anti‐CPG_2_ primary antibody raised in rabbit diluted 1/1000 in 1% milk proteins/PBS (wt/vol) for 1 hr at room temperature, followed by incubation for 1 hr at room temperature with anti‐HRP diluted 1/1000 in 1% milk proteins/PBS (wt/vol). Immunoreactive detection of MFE, fragment of the MFECP1 protein was preformed by incubation with polyclonal anti‐MFE primary antibody raised in rabbit diluted 1/1000 in 1% milk proteins/PBS (wt/vol) for 1 hr at room temperature, followed by incubation for 1 hr at room temperature with anti‐HRP diluted 1/1000 in 1% milk proteins/PBS (wt/vol). His_6_ on the MFECP1 protein was detected with mouse anti‐His_4_ monoclonal antibody (Qiagen Ltd, Manchester, UK) diluted 1/1000 in 1% milk proteins/PBS (wt/vol) for 1 hr at room temperature, followed by incubation for 1 hr at room temperature with sheep anti‐mouse monoclonal anti‐HRP (GE Healthcare, Hatfield, UK) diluted 1/500 in 1% milk proteins/PBS (wt/vol). Final staining of all western blots was achieved by incubation with 0.25 mg/ml 3,3′‐diaminobenzidine with H_2_O_2_ (1/2,000). Washing steps consisted of five washes with 0.1% Tween^®^ 20/PBS (vol/vol) followed by three with PBS.

#### 2.8.4 Fusion protein sample preparation

All fusion protein stock solutions used in shear experiments were prepared in a 0.01 M PBS solution at pH 7.4, 0.138 M NaCl; 0.0027 M KCl made up with ultrapure deionised water.

#### 2.8.5 USD experimental design

The MFECP1 fusion protein was subjected to controlled levels of shear in an USD rotating disk shear device previously described in Boychyn et al. (2001) and Levy et al. (1999). The device was fabricated from 316 stainless steel in house (Mechanical Engineering Workshop, UCL, London, UK), comprising of a rotating disc housed inside a shear chamber. The dimensions of the rotating disk were as follows; radius of disk = 0.0400 M, thickness of disk = 0.0015 M. The disk was attached to a 7.2V 500BB race VS Motor (Graupner, Henriettenstr, Germany) by a stainless steel shaft through a polytetrafluoroethylene (PTFE) seal. The dimension of the internal shear chamber was: diameter = 0.0500 M, height = 0.0100 M, holding a total protein volume of 20 ml. During shearing over a 1‐hr period temperature of the internal chamber was monitored with a 1‐mm PTFE protected type (T) thermocouple (RS Components, Ltd., UK) which was attached to a model 2006T, temperature reader (RS Components, Ltd.). The temperature of the solution was maintained at 4°C throughout the experiment with an ice cooled water bath. An internally built tachometer monitored the speed of the rotating disk maintaining constant revolutions per minute (rpm) typically for all shear experiments described here this was at 5,000 rpm speed.

Protein stock solutions ~500 ng/ml were sheared for 1 hr at 5,000 rpm at a constant temperature and duplicate 100 µl samples were taken at 0‐, 300‐, 600‐, 1,200‐, 1,800‐, 2,400‐, 3,000‐, 3,600‐s intervals and measure on ELISA immediately after shearing.

Samples from the 100 µg/ml, shear experiment were assayed by ELISA, enzyme activities, SDS‐PAGE gel and western blot later. One hundred and eighty microlitre of sheared protein sample was taken from the shear device at time points over a 1‐hr period as previously described. Sheared samples (180 µl) with 0.01% F68 were diluted with 20 µl of PBS then frozen down into 4 × 50 µl aliquots for analysis. Sheared samples (180 µl) without 0.01% F68 were diluted with 20 µl of 0.1% F68 (vol/vol then frozen down into 4 × 50 µl aliquots for analysis later. This controlled procedure compensated for any false positive effects the reagent may have given during analysis.

Figures 6 and 7 show *C*/*C*_0_ the concentration fraction of the intact MFECP1 fusion protein present, where, *C* is the concentration of protein in ng/ml at time *t* and *C*_0_ the initial concentration of protein in ng/ml at *t* = 0. The errors bars show the range between each duplicate, 100 µl samples. A first‐order kinetic relationship, *C*/*C*_0_ = *a* + *be* ^−^ *^kt^*, was used to fit the data and generate the degradation rate constants (*k*_1_). Figures 8 shows *E*/*E*_0_ the concentration of active enzyme present, where, *E* is the activity of the CPG_2_ enzyme in U/ml at time *t* and *E*_0_ = initial activity of the CPG_2_ enzyme in U/ml at time *t* = 0.

#### 2.8.6 Air–liquid interface

A 50% and 0% air–liquid interface was applied to MFECP1 fusion protein stock solution to assess protein robustness. A 50% air–liquid interface indicated the shear device was filled with 10 ml of protein solution (half full) and 0% air–liquid refers to the device being filled to 20 ml (full capacity).

#### 2.8.7 Data analysis curve fitting

This study used the nonlinear fit functions found in SigmaPlot 9.0 (SSI, CA) to fit the experimental data points and generate the degradation constants. The nonlinear regression method used by SigmaPlot was based on the Levenberg–Marquardt least square fitting algorithm.

## 3 RESULTS

### 3.1 Large‐scale activity levels of MFECP1 fusion protein

**Figure 1:**
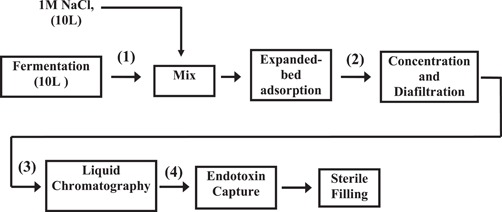
Process flow sheet of the full‐scale production and purification of the fusion protein (MFECP1). Samples were taken after the following stages of the purification chain and analysed for enzyme activities: fermentation harvest (1); EBA capture and release (2); membrane concentration and diafiltration (3); liquid chromatography (4). EBA: expanded‐bed adsorption

**Figure 2:**
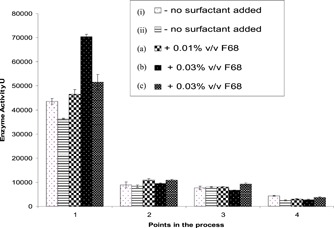
Comparison of the enzyme CPG_2_ activity taken after different stages of a full‐scale production run (10 L fermenter working volume): (1) fermentation harvest; (2) EBA capture and release; (3) membrane concentration and diafiltration; (4) liquid chromatography. Fermentations were conducted without and with F68 (see legend). In fermentation (a) a single dose equivalent to 0.01% of the broth was added at the start of fermentation. For (b) and (c) three doses equivalent to 0.01% of the broth volume were made during the course of the fermentation. The total activities given are the mean of three measurements ±*SD*. EBA: expanded‐bed adsorption

Figure 1 shows the process flow sheet of the facility used to produce and purify the fusion protein (MFECP1) at a large scale. The activity of the CPG_2_ enzyme was monitored during bioprocessing, giving an approximate measure of the levels of MFECP1 fusion protein lost during production and purification. It was observed that significant losses were occurring early on during the bioprocessing, from harvest to EBA capture (Figure 2) and progressive losses further down the purification train. The activities measured after EBA capture was primarily intact His‐tagged positive protein, hence the losses from the harvest step to the EBA capture step could be attributed to shear related degradation of the His‐tag from the MFECP1 protein, proteolytic damage and denaturation of the enzyme. However, protein losses can be expected throughout the bioprocessing due to harsh bioprocessing. A series of complex events were thought to be occurring these include aggregation, surface attachment and protein breakdown.

**Figure 3:**
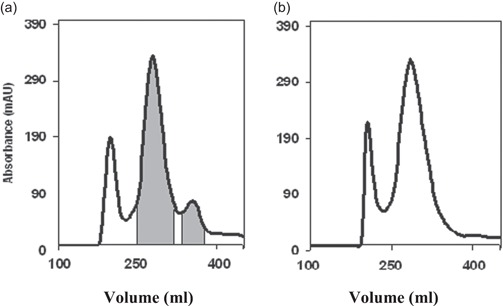
Eluate of protein purified on liquid chromatography from fermentations treated with and without F68. The protein produced from fermentation (i), no F68; protein produced from fermentation (ii), plus 0.03% of F68 (vol/vol). Fusion protein yield peak is normally found from 250 to 300 ml, fusion protein breakdown is found between 350 and 400 ml. The first peak 150–200 ml is methanol induced by product of the cells. Concentrated and the diafiltered fusion protein was analysed by liquid chromatography. Twenty millilitre of protein was applied to a Superdex 200 column at 0.4 ml/min, equilibrated with filtered PBS mobile phase 0.5 hr before loading. Profile in Figure 3b shows the pooling strategy for further detailed protein analysis (see Figure 4). PBS: phosphate‐buffered saline

The addition of F68 to the large‐scale fermentation of MFECP1 was conducted to observe any beneficial effects the surface active agent might have had on the protein integrity and therefore yield. The results in Figure 2 show that no discernable trends in activity measurements were observed between fermentations. However, Figure 3 shows the addition of the agent improved the profile of the protein coming off the last downstream purification stage (liquid chromatography), breakdown of the fusion protein can be seen in Figure 3a,b, where it was seen that the fermentation treated with surfactant produced less breakdown product (peak 350–400 ml; Figure 3b).

**Figure 4:**
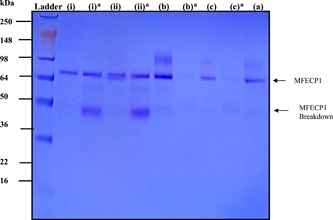
Pooled protein fractions from the liquid chromatography eluate and effect of F68 in the original fermentation broth. The pooled fractions are from the eluate volumes from 250 to 300 ml (main fusion protein peak) and 350–400 ml (secondary peak), see Figure 3 (b). Lanes shown are: M, molecular weight markers 250–16 kDa; (i, i)*, fermentation (i) no F68, main and secondary peaks respectively; (ii, ii)*, fermentation (ii) no F68, main and secondary peaks respectively; (b, b)*, fermentation (b) 0.03% F68, main and secondary peaks respectively; (c, c)*, fermentation (c) 0.01% F68, main and secondary peaks respectively; (a), fermentation 0.01% F68, main and (a)*, secondary peak was analysed on a separate gel showing no breakdown peak (data not shown) [Color figure can be viewed at wileyonlinelibrary.com]

**Figure 5:**
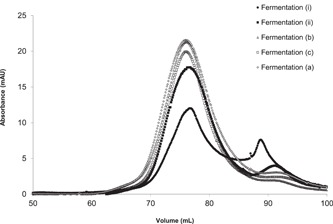
Purified diafiltered protein analysed on liquid chromatography from fermentations treated with and without F68. The protein produced from fermentation (i, ii), no F68; a protein produced from fermentation (a), plus 0.01% of F68; protein produced from fermentations (b) and (c) plus 0.03% of F68 (wt/vol). Fusion protein yield peak is normally found from 75 to 85 ml, fusion protein breakdown is found between 85 and 95 ml. Concentrated and the diafiltered fusion protein was analysed by liquid chromatography. One millilitre of protein was applied to a Superdex 200 column at 0.4 ml/min, equilibrated with filtered PBS mobile phase 0.5 hr before loading. PBS: phosphate‐buffered saline

Confirmation that the addition of F68 to the fermentations reduced the breakdown of the fusion protein can be seen in Figure 4. The band normally found at 50 kDa showing broken fusion was not observed in the analysis of liquid chromatography profiles from fermentation treated with F68. Further analysis of the pooled protein fractions taken after liquid chromatography can be seen in Figure 5. Here results showed that the fraction of breakdown normally found at 85–95 ml decreased when F68 was present in the fermentation. Results also show that the addition of the agent reduced the batch to batch variation of the main fusion protein peak area.

### 3.2 Small‐scale processing effects

As a result of these findings, the small‐scale processing effects of the fusion protein at USD were investigated.

#### 3.2.1 The effect of air–liquid interfaces on MFECP1 protein integrity

**Figure 6:**
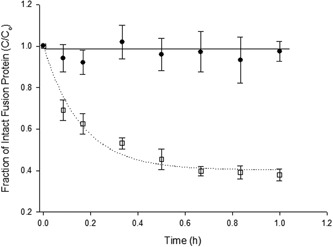
The effect of air–liquid interface and shear on the loss of fusion protein. Experimental conditions were: (^__^•^__^), *C*_0_ = 476 ng/ml, no air–liquid interface, 5,000 rpm and (^…^⎕^…^), *C*_0_ = 450 ng/ml, 50% air–liquid interface, 5,000 rpm; *C*_0_ = initial concentration of intact MFECP1 fusion protein as measured by ELISA. All shear experiments were conducted at a constant temperature of 4°C maintained by a cooled water bath. Data points show the mean value and range of duplicate samples. Curves are lines of best fit for a first‐order kinetic relationship to an equilibrium (nonzero value). ELISA: enzyme‐linked immunosorbent assay

Air–liquid interface was applied to a fusion protein solutions ~500 ng/ml to assess protein robustness. It was found that a 50% vol/vol air–liquid interface with a constant shear condition of 5,000 rpm (rotational disk speed) was detrimental to the integrity of the MFECP1 fusion protein in the USD shear device (Figure 6). The results here show that 60% of the initial MFECP1 protein was lost after 0.5 hr of shearing giving a first‐order *k*_1_ constant of 3.8 (±0.49) and a final equilibrium value of 133 ng/ml. However, 1 hr of shear (5,000 rpm) with no air–liquid interface resulted in <2% of the protein being lost, giving a final concentration of 469 ng/ml and a constant of zero.

#### 3.2.2 The effect of F68 on MFECP1 protein integrity

It was found that both Pluronic^®^ F68 0.01% vol/vol and antifoam (organic solution), 0.01% vol/vol reagents reduced the extent of MFECP1 loss when added to MFECP1 protein solutions before shearing. Comparison of the first‐order constants (*k*_1_) shows how reagents like F68 and antifoam reduced the rate of MFECP1 loss over 1 hr of shearing. The control experiment showed that fusion protein without agents decreased at a rate of 8.7 (±0.57). However, when F68 was added this rate was reduced to 0.49 (±0.1840). The addition of antifoam and F68 gave a lower rate of loss, improving the level of the final equilibrium concentration after 1 hr of shearing. Antifoam was tested because the literature suggested that F68 protected biological products by producing foam (Chattopadhyay et al., 1994) or coating air bubbles (Michaels et al., 1990); however antifoam was present during the fermentation, so it was important to prove that this did not compromise the protective effect F68 had on MFECP1 by reducing the amount of foam present at USD.

Next, we assessed the effect of F68 at higher concentrations of fusion protein typically 100 µg/ml, approximately the amount of protein found in the fermenter. The effect of F68 on fusion protein integrity can be seen in Figures 8, 9, 10. The presence of F68 reduced the rate of enzyme aggregation over a 1‐hr period (Figure 8). The rate of enzyme loss was reduced from 1.29 (±0.44) to apparently zero, hence improving the measured CPG_2_ enzyme activity after shearing. Small‐scale shear experiments conducted with 100 µg/ml of fusion protein showed that the addition of F68 reduced the amount of fusion protein breakdown product (Figure 9). These results suggested that F68 might have a stabilising or protective effect on the protein during shear conditions.

Figure 10 demonstrated the stabilising effect the addition of the F68 detergent had on the total protein stained by coomassie blue on an SDS‐PAGE gel. Fusion protein samples were analysed after being sheared for 1 hr with a 50% air–liquid interface with and without F68. The results here show that when the protein was sheared in the presence of F68 an intense band around 70 kDa, the approximate size of the fusion protein was detected. Control experiments showed that protein sheared for 1 hr, without F68, produced a band of 70 kDa, at 0 hr, however, this faded quickly over the shearing time resulting in a lighter band intensity after 1 hr. Western blot analysis of the same sheared samples uncovering the positive immunoreactivity of His6 (Figure 10b). The western shows the same effect as seen with the SDS gel. Figure 10b showed that when F68 was present a significantly higher immunoreactive intensity was present than without over the shearing time.

## 4 DISCUSSION

The work presented here demonstrates that detailed characterisation of small quantities of sometimes very precious protein solution could be used to characterise protein loss during large‐scale bioprocessing. The importance of this study centres on using millilitres of dilute material to quickly generate very useful data, early on in a bioprocess development stage. This can then be translated to the large‐scale production, improving yields and saving time, money and labour (Kolade et al., 2015; Rayat et al., 2016).

The results showed how susceptible a fusion protein MFECP1 was to the harsh large‐scale bioprocess conditions encountered during production and purification, giving only a 10% enzyme activity recovery yields from a 10‐L fermentation broth. This is not uncommon in the bioprocess field as a large number of complex biopharmaceuticals have been shown to be degraded or deactivated by high shear effects during processing (Thomas & Geer, 2011). This investigation focused on the integrity of a biopharmaceutical antibody‐fusion protein used in a two‐phased drug delivery system to treat colorectal carcinoma (Bagshawe, 1989; Michael et al., 1996). Hence it was imperative that the MFECP1 fusion protein was not degraded or deactivated in anyway which would otherwise result in loss of therapeutic activity (Kolade et al., 2015). A USD shear device was used to mimic the harsh bioprocessing conditions interacting with the protein during production and purification. This device analysed how robust the MFECP1 antibody‐fusion protein was to a variety of bioprocessing parameter that exacerbated its rate of protein loss (Biddlecombe et al., 2007). It was found that the combination of air–liquid interface and a rotational shear field of 5,000 rpm was particularly detrimental to an MFECP1 protein solution. Sixty percent of fusion protein solutions (around ~500 ng/ml) were lost in an air–liquid interface (no air in the system had little effect on the degradation of the MFECP1 protein). Protein loss in the presence of an air/liquid interface has been previously observed for dextransucrase (Lencki et al., 1993), and human/bovine serum albumins (Oliva, Santoveña, Fariña, & Llabrés, 2003), so our data here are consistent with other published work (Kolade et al., 2015).

The literature also highlights ways in which high shear inflicted on bioprocess materials can be reduced. One such way is by the addition of detergents like Pluronic^®^ F68. The addition of F68 to mammalian cell cultures can reduce the high shear effects on the fragile mammalian cells encounter during production and purification (Chattopadhyay et al., 1994; Michaels et al., 1990; Tait et al., 2012). The most common detergent F68 was added to the large‐scale fermentation media to observe any potential beneficial trends.

Samples taken throughout the fermentation run and downstream processing were analysed for biomass and enzyme activities. The results showed that the addition of F68 to the fermentation media had little effect on the biomass yield as predicted from the small‐scale shaker flask experiments. There was no notable increase in enzyme activity (Figure 2). However, the addition of F68 did improve the protein peak profile after liquid chromatography (Figures 3 and 5), producing less breakdown and a more homogenous product. It is thought that the F68 may be reducing the amount of autocatalysis of the product leading to a significantly higher yield (Figures 3 and 4) showing an increase of intact fusion protein with little breakdown products formed. SDS gel analysis (Figure 4) confirmed that less breakdown was present when F68 was added showing that the agent may have a protective effect upon the fusion protein during bioprocessing. It was thought early parts of the process benefited from the effect of F68, as this was due to the very high concentration of the surfactant in the process solution, resulting in lower surface shearing effects and inhibition of autocatalysis and/or protease attack in bioreactor process solution. Also, a high proportion of the F68 should have been removed at EBA; therefore, downstream process improvements seen could be due to reducing breakdown of the product before EBA and or reducing coelution of products that could breakdown the fusion protein.

**Figure 7:**
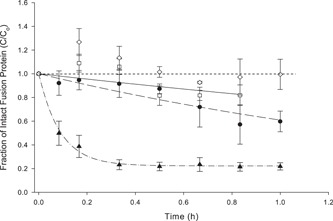
The effect of surface active agents on the loss of fusion protein in the presence of air–liquid interface. Fixed shear conditions were: 50% air–liquid interface and 5,000 rpm for all ultra‐scale‐down experiments. Initial concentrations and reagents added were: (^_._._.^▲^_._._.^), *C*_0_ = 554 ng/ml, no added surface active agents; (– –•– –), *C*_0,_= 583 ng/ml, plus 0.01% F68 (vol/vol); (—⎕—), *C*_0_ = 680 ng/ml plus 0.01% antifoam (vol/vol); (‐‐‐◊‐‐‐), *C*_0_ = 690 ng/ml plus 0.01% F68 (vol/vol) and 0.01% antifoam (vol/vol). All shear experiments were conducted at a constant temperature of 4°C maintained by a cooled water bath. Data points show the mean value and range of duplicate samples. C_o_ = initial concentration of intact MFECP1 fusion protein as measured by ELISA. Points with a ±20% error outside the calibration range were excluded. Curves are lines of best fit for a first‐order kinetic relationship, to an equilibrium (nonzero value). ELISA: enzyme‐linked immunosorbent assay

The characterisation of the results seen on a large scale were attempted by using the USD studies. The USD experiments showed that the presence of a 50% air–liquid interface increased the rate of fusion protein loss (Figure 6). It was also found that F68 reduced the rate of protein loss over a 1‐hr period; whereas in the control experiments the protein had been degrading. It is thought Pluronic F68 may be reducing the surface adsorption of the protein leading to possible denaturation and aggregation effects (Emoto, Malmstena, & Van Alstineabc, 1998). The surface absorption effect of F68 may also be increasing the amount of protein available in free solution thus increasing the rate of protease–carboxypeptidase degradation. Other reagents were also identified that stabilised the fusion protein, for example, antifoam; these reagent were already involved in the large‐scale production media so were not investigated further (Figure 7).

**Figure 8:**
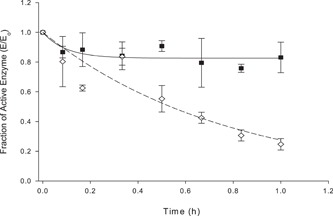
The fraction of enzyme activity recovered from sheared samples (*E*_0_ = initial enzyme activity), data points show the mean of three values; error bars are equal to ±*SD*, curves are lines of best fit for a first‐order kinetic relationship to an equilibrium (nonzero value). All shear experiments were conducted at a constant temperature of 4°C maintained by a cooled water bath. The MFECP1 protein used in this shear experiment was His‐tag purified

**Figure 9:**
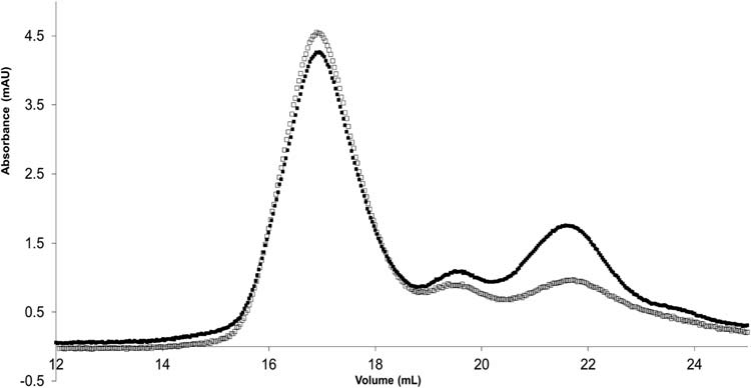
The effect of F68 on fusion protein breakdown during 1 hr of shear at 5,000 rpm with a 50% air–liquid interface. 100 μg/ml of the fusion protein was analysed by FPLC from USD shear experiment. (a) No detergent added: (⎕), 0 hr; (•), 1 hr. (b), 0.01% F68 (vol/vol) added: (⎕), 0 hr; (•), 1 hr. The traces show that protein normally found between 15 and 20 ml, produced a breakdown peak found at 20–25. Sheared samples were analysed on a 15‐ml small‐scale liquid chromatography column (Superose^TM^ 6 10/300 GL; GE Healthcare) with PBS as mobile phase at 0.4 ml/min flow rate. All shear experiments were conducted at a constant temperature of 4°C maintained by a cooled water bath. FPLC: fast protein liquid chromatography; PBS: phosphate‐buffered saline; USD: ultra‐scale down

**Figure 10:**
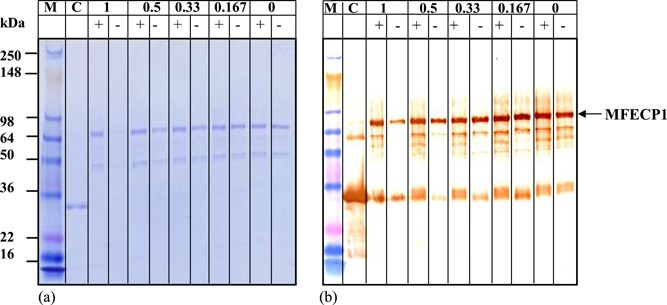
The effect of F68 during fixed shear conditions, 5,000 rpm, 50% air–liquid interface as measured on SDS‐PAGE gel electrophoresis and western blots. MFECP1 fusion protein solution, C_o_ = 100 μg/ml, was sheared over a 1‐hr period and samples were analysed on (a) SDS‐PAGE gel (b) anti‐MFE. For gel and all western blots samples: M, molecular weight marker 250 to 16 kDa; C, controls MFE and CPG_2_ fragment proteins; +, the addition of 0.01% F68 (vol/vol); −, no reagent. Note all sheared samples without 0.01%, vol/vol F68 were spiked with appropriate amounts of F68 before analysis to compensate for any false positive effects the reagent may have given during analysis. SDS‐PAGE: sodium dodecyl sulfate‐polyacrylamide gel electrophoresis [Color figure can be viewed at wileyonlinelibrary.com]

Although F68 seemed to be protecting the protein loss at low concentrations around ~500 ng/ml. The bioreactor handled protein concentrations 200 times greater around 100 µg/ml. Therefore the protective effect of F68 on MFECP1 fusion protein had to be verified at this concentration. The results from Figures 8 and 10 showed that F68 reduced the loss the fusion protein over a 1‐hr period; however, the effect was not as prominent as in the lower concentration shear experiments. Western blot and SDS gel analysis showed that the detergent hand a beneficial effect on protein loss (Figure 10b). Results from the same shear experiments showed that the enzyme activity was not lost when F68 was added to the protein solution before shearing (Figure 8). The apparent breakdown product of fusion protein was also reduced at USD (Figure 9). Further analysis of Figures 9 and 10 shear experiments showed that the addition of 0.01% vol/vol F68 to the process solution reduced the detectable fusion protein. It is thought that F68 may have stabilised the fusion protein, resulting in a higher amount being detected during analysis. In conclusion, the USD data conducted with protein at 100 µg/ml showed that F68 might reduce fusion protein loss showing that the small effects seen on a large scale could have been predicted with small‐scale studies.

## 5 CONCLUSION

In conclusion, the addition of F68, a shear protectant to the fermentation media had little effect on the overall enzyme activity throughout the process. However, it was observed that the profile of the final product had changed resulting in lower impurity in the final product. Autocatalysis may have been one of the primary causes for fusion protein loss at the early stages and further along the process train. The results do show a significant improvement in the bioprocessing of the fusion protein, whether this was due to shear protection or reduction of autocatalysis breakdown are aspects to cover in future research. The addition of Pluronic might reduce mAb denaturation during fermentation and downstream processing so that co‐elution enzymes (carboxypeptidases) are less able to destroy the target fusion protein. It has been shown that USD experiments could have indicated parameters that exacerbate the rate of fusion protein loss and identify surfactants that protect this loss. This study shows that detailed characterisation of very small quantities of protein solution can be used to improve the bioprocessing, resulting in time saved during large‐scale process development. If the USD can be harnessed earlier on in the production of biopharmaceuticals, it could save the potential biotech industry larger amounts of time, labour, and capital cost. Hence, resulting in the ability to dominate a patent for a longer period generating exclusivity in the market place.
